# Approximate solutions for HBV infection with stability analysis using LHAM during antiviral therapy

**DOI:** 10.1186/s13661-020-01373-w

**Published:** 2020-04-16

**Authors:** M. Aniji, N. Kavitha, S. Balamuralitharan

**Affiliations:** 1 Department of Mathematics, University College of Engineering, Rajamadam, Pattukkottai, Tamilnadu, India; 2 Department of Mathematics, Faculty of Engineering and Technology, SRM Institute of Science and Technology, Kattankulathur, Tamilnadu, India

**Keywords:** 34G20, 34A34, Antiviral therapy, LHAM, Mathematical modeling

## Abstract

Hepatitis B virus (HBV) is a life-threatening virus that causes very serious liver-related diseases from the family of Hepadnaviridae having very rare qualities resembling retroviruses. In this paper, we analyze the effect of antiviral therapy through mathematical modeling by using Liao’s homotopy analysis method (LHAM) that defines the connection between the target liver cells and the HBV. We also examine the basic nonlinear differential equation by LHAM to get a semi-analytical solution. This can be a very straight and direct method which provides the appropriate solution. Moreover, the local and global stability analysis of disease-free and endemic equilibrium is done using Lyapunov function. Mathematica 12 software is used to find out the solutions and graphical representations. We also discuss the numerical simulations up to sixth-order approximation and error analysis using the same software.

## Introduction

Hepatitis B virus (HBV) infection is one among the very serious infections that threatens the people worldwide, less than 300 million people live with chronic hepatitis B infection worldwide. It directly or indirectly affects the liver, causing diseases affecting also infants and young children. This virus is genetically from the family of Hepadnaviridae having very rare qualities resembling retroviruses [1]. Hepatitis B is a viral infection that attacks the liver and can cause both acute and chronic diseases. The infection is a potentially life-threatening liver infection caused by the hepatitis B virus (HBV). In 2015, hepatitis B resulted in an estimated 887,000 deaths, mostly from cirrhosis and hepatocellular carcinoma. As of 2016, 27 million people (10.5% of all people estimated to be living with hepatitis B) were aware of their infection, while 4.5 million (16.7%) of the people diagnosed were on treatment. Hepatitis B can be prevented by vaccines that are safe, available, and effective. The modalities of transmission of the HVB are completely different from those of the HAV (hepatitis A virus) but can be compared to those of the HCV and HIV virus. HCV is most commonly transmitted through contact with blood or other body fluids as well as from a positive mother to a child during birth and delivery. This poses a major public health problem for the protection of those living with affected patients, healthcare professionals and requires serious and effective control of blood transfusions. Transmission of the virus may also occur through the reuse of needles and syringes either in healthcare settings or among persons who inject drugs [2–4]. In addition, infection can occur during medical, surgical, and dental procedures, through tattooing, or through the use of razors and similar objects that are contaminated with infected blood. HBV infection can be either acute or chronic. The illness can be asymptomatic, symptomatic and may progress to cirrhosis. There is no specific treatment for acute hepatitis B. Chronic HBV infection is defined as persistence of hepatitis B surface antigen for six months and more after acute infection with HBV. It is a major public health problem because the majority of people are unaware of their HBV infection; they are asymptomatic and can transmit the disease. Chronic hepatitis B infection can be treated with medicines, including oral antiviral agents. Only a proportion (estimates vary from 10% to 40% depending on the setting and eligibility criteria) of people with chronic hepatitis B infection will require treatment. So, to prevent this disease and infection, hepatitis B vaccine should be taken. The vaccine is effective to prevent the infection increasing the protective levels of antibodies [5, 6]. Mathematical model is an essential tool to understand the virus dynamics, and it intensifies our understanding of the virus. The first important mathematical models covering immune response were developed by Anderson and May on infectious diseases of humans, in particular HIV and other viral diseases [7]. Other authors proposed different mathematical models concerning the transmission of viral infection. Blessing proposed a mathematical model of hepatitis B virus transmission dynamics with considerations of different classes of individuals, namely immunized, susceptible, latent, infected, and recovered classes [8]. Kamyad et al. suggested a mathematical model considering the immune response of vaccinated infants, what treatments given to an infected person, and how it controls the transmission of HBV [9]. The humoral immune response is also important in HCV infection and is the key to vaccine development [10]. Su, B. et al. [11] proposed an HBV transmission model utilized to know the vaccination effectiveness and transmission control.

Application of the mathematical models to different parameters shows that some patients get cleared of the virus quickly due to drug therapy, whereas in case of other patients the drug therapy works much slower [12]. Therefore, in some cases treatment has to be stopped. Then, the viral degeneration is observed in most patients [13]. In this paper, we try to get a deeper understanding of the complex virus dynamics that is seen during drug therapy in HBV infections. Such knowledge helps to improve the treatment by informing what drugs to use, when to start, and when to stop [14–17]. The main motivation of the paper is that this will help the young researchers of medicine as well as science find a solution comparing to real world problems pertaining to HBV antiviral therapy. Finding the analytical solutions for this model is very challenging. But we found the analytical solution for Eq. (2.1) using the LHAM method. Our model is very useful and it is easy to find the analytical solutions. We also can find the numerical simulations by using MATLAB for the same equations.

This paper is divided into eight parts. The first part of the paper forms an introduction which deals with the existing literature and proposed work. The second and third parts talk about mathematical modeling and analytical study of the HBV model. In these parts we analyze the local and global stability of HBV. The fourth part explains the LHAM method, and the fifth part is applications that have been used to find out the solutions using LHAM. The sixth part of the paper contains numerical experiments which deal with numerical simulations obtained up to sixth-order approximations using Mathematica 12 software. Error analysis forms the seventh part of the paper, the eighth part is discussion, and final part of the paper is conclusion.

## Mathematical modeling

We consider the mathematical model for the basic virus dynamics [18]. This model represents an in-host model for the interaction between liver cells (uninfected and infected) and the virus, which is derived from the mathematical model. Here, *X* represents the target uninfected cells (uninfected hepatocytes), *Y* represents the infected cells (infected hepatocytes), and *Z* represents the HBV virus. This model represents the target cells that are infected at rate *β*, and infection occurs by contact between the target cells and the virus. It also considers that the infected cells die at a rate of *δ* and production of new virus happens at a rate of *v*. The rate of dead virus is *c*. The new target cells introduced in the liver may die before getting infected. The constant production rate is denoted by *s*, and the natural death rate is denoted by $d_{T}$. Parameters and variables are presented in Table 1 [19]. The variables are considered either horizontal (through contact with infective individuals) or vertical transmission (directly from the parents). The horizontal transmission is less efficient, but the vertical transmission occurs with great frequency (70% to 100%) when the mother has acute hepatitis B during delivery.

**Table 1:** Values of the parameter model

Parameter	Explanation	Values
*s*	production of constant hepatocyte	1 cell day^−1^ mL^−1^
$d_{\mathrm{T}}$	death rate of hepatocyte	0.01–9 × 10^−4^ day^−1^
*β*	rate of infectivity	1 × 10^−10^–6.6 × 10^−8^ mL virions^−1^ day^−1^
*δ*	infected hepatocytes killing rate	0.06–0.25 day^−1^
*ρ*	rate of recovery	0 day^−1^
*v*	virus production	1.4–164 virions cell^−1^ day^−1^
*c*	virus clearance	0.18–1 day^−1^
*η*	effect of therapy in blocking infection	0.2–0.5
*ε*	effect of drug in blocking new virus production	0.99934–1

These assumptions lead to the following model:

2.1 $$ \begin{aligned}& \frac{dX}{dt} = s - (1 - \eta )\beta ZX - d_{T}X + \rho Y, \\ &\frac{dY}{dt} = (1 - \eta )\beta ZX - \delta Y - \rho Y, \\ &\frac{dZ}{dt} = (1 - \varepsilon )vY - cZ. \end{aligned} $$

The initial and boundary conditions of finding the solution of Eq. (2.1) are

$$\begin{aligned}& X(0) = 0;\qquad Y(0) = 0;\qquad Z(0) = 0; \\& X(0) = 10^{8};\qquad Y(0) = 10^{-2};\qquad Z(0) = 10. \end{aligned}$$

**Figure 1:**
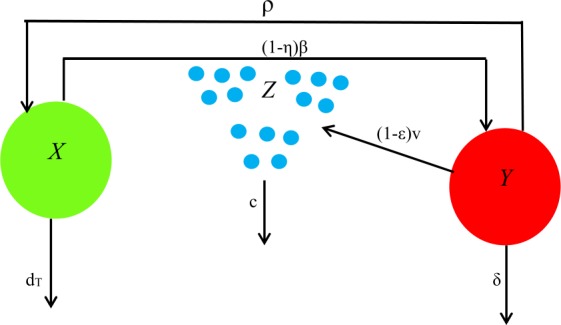
Diagram for basic virus dynamics model

We consider three cells as the target cells, the infected cells, and the hepatitis B virus cells [20]. This model defines the interaction between the target liver cells and the HBV [21]. The HBV gets cleared for strong immune response that cures cells and makes them not to get reinfected. All parameter estimations are obtained in Table 1. Also, it represents the efficacy of the treatment on blocking the production of virion [9], and compartmental diagrams are given in Fig. 1.

## Analytical study of the HBV model

### Boundedness and positivity

#### Theorem 3.1

*Let*$A:[0, +\infty ] \rightarrow B^{3}$. *If*$A(0) \in B_{ +}^{3}$*then*$A(t) \in B_{ +}^{3}$ ∀*tin*$[0, + \infty ]$, *then the solution is*$A(t) = (X,Y,Z)$.

To prove this for $t > 0$, $\dot{X} \vert _{X = 0}$, $\dot{Y} \vert _{Y = 0}$ and $\dot{Z} \vert _{Z = 0}$ are positive. Therefore, the solutions of model (2.1) are positive.

#### Theorem 3.2

*Let*$A = \{ ( X,Y,Z ) \in B_{ +}^{3}|0 \le X + Y + Z \le \bar{X}_{0},X,Y,Z \ge 0 \} $, *where*$\bar{X}_{0} = \frac{s}{d_{T}}$. *The solutions of system* (2.1) *are bounded in a compact set*. *Obviously*, *the solutions of* (2.1) *are bounded in the region A*.

### Equilibrium analysis

Disease-free equilibrium and endemic equilibrium

$$\begin{aligned}& P^{0} = (X_{0},Y_{0},Z_{0}) = ( \bar{X}_{0},0,0 ), \\& P^{*} = \bigl(X^{*},Y^{*},Z^{*} \bigr), \\& X^{*} = \frac{c(\delta + \rho )}{\beta v(1 - \varepsilon )(1 - \eta )}, \\& Y^{*} = \frac{1}{\delta } \biggl[ s - \frac{cd_{T}(\delta + \rho )}{\beta v(1 - \varepsilon )(1 - \eta )} \biggr], \\& Z^{*} = \frac{1}{\delta } \biggl[ \frac{s\beta v(1 - \varepsilon )}{c} - \frac{d_{T}(\delta + \rho )}{(1 - \eta )} \biggr]. \end{aligned}$$

### Basic reproduction number

$$ U = \begin{bmatrix} 0 & \frac{\beta s(1 - \eta )}{d_{T}} \\ 0 & 0 \end{bmatrix}\quad \text{and}\quad V = \begin{bmatrix} \delta + \rho & 0 \\ - v(1 - \varepsilon ) & c \end{bmatrix}. $$

Therefore,

$$UV^{ - 1} = \begin{bmatrix} \frac{s\beta v(1 - \eta )(1 - \varepsilon )}{cd_{T}(\delta + \rho )} & \frac{\beta s(1 - \eta )}{cd_{T}} \\ 0 & 0 \end{bmatrix}. $$

Hence, by [22],

$$ R_{0} = \frac{s\beta v(1 - \eta )(1 - \varepsilon )}{cd_{T}(\delta + \rho )}. $$

When $R_{0} < 1$ the virus vanishes, otherwise it continues. Since the antiviral therapy is given, $(\delta + \rho )$ will never be zero.

### Stability analysis

#### Proposition 3.3

$P^{0}$*is locally asymptotically stable for*$R_{0} < 1$*and unstable otherwise*.

#### Proof

$$\begin{aligned}& J = \begin{bmatrix} - ((1 - \eta )\beta Z + d_{T}) & \rho & - (1 - \eta )\beta X \\ (1 - \eta )\beta Z & - (\delta + \rho ) & (1 - \eta )\beta X \\ 0 & (1 - \varepsilon )v & - c \end{bmatrix}, \\& J\bigl(P^{0}\bigr) = \begin{bmatrix} - d_{T} & \rho & \frac{ - (1 - \eta )\beta s}{d_{T}} \\ 0 & - (\delta + \rho ) & \frac{(1 - \eta )\beta s}{d_{T}} \\ 0 & (1 - \varepsilon )v & - c \end{bmatrix}. \end{aligned}$$

Therefore the eigenvalues of the disease-free equilibrium are $- d_{T}$, $- (\delta + \rho )$, and −*c*.

Eigenvalues at disease-free equilibrium are all negative, and hence it is stable. Therefore if $R_{0} < 1$, $P^{0}$ is locally asymptotically stable. □

#### Proposition 3.4

$P^{0}$*is globally asymptotically stable for*$R_{0} \le 1$*and unstable otherwise*.

#### Proof

Let the Lyapunov function $L(X,Y,Z):R_{ +}^{3} \to R_{ +}^{3}$ be defined as

3.1 $$ L(X,Y,Z) = \frac{(1 - \varepsilon )vY}{(\delta + \rho )} + Z. $$

Differentiating (3.1) with respect to “t”, we have

$$\begin{aligned}& \begin{aligned}[b] \dot{L}& = \frac{(1 - \varepsilon )v}{(\delta + \rho )} \bigl[ (1 - \eta )\beta ZX - ( \delta + \rho )Y \bigr] + (1 - \varepsilon )vY - cZ \\ &\le \frac{\beta vs(1 - \varepsilon )(1 - \eta )Z}{d_{T}(\delta + \rho )} - cZ, \end{aligned} \end{aligned}$$

since

$$\begin{aligned}& \bar{X}_{0} = \frac{s}{d_{T}} \ge X, \\& \dot{L} \le cZ(R_{0} - 1), \end{aligned}$$

which implies $\dot{L} \le (R_{0} - 1)Z \le 0$. Therefore $\dot{L} = 0$ only when $Z = 0$ in (2.1) such that $X \to \frac{s}{d_{T}}$ and $Y \rightarrow 0$ as $t \rightarrow \infty $. Hence, by [23], $P^{0}$ is globally asymptotically stable when $R_{0} > 1$.

Stability analysis has been discussed in this study, which gives the stable equilibrium points obtained from the characteristic equation systems of differential equations. The type of an equilibrium point is determined by the eigenvalues of the Jacobian matrix. Stability analysis is obtained by determining the eigenvalues of the Jacobian matrix around equilibrium points. □

#### Proposition 3.5

$P^{*} = (X^{*},Y^{*},Z^{*})$*is locally asymptotically stable for*$R_{0} > 1$*and unstable otherwise*.

$$\begin{aligned}& J\bigl(P^{*}\bigr) = \begin{bmatrix} - \beta ( \frac{s\beta v(1 - \varepsilon )(1 - \eta )}{\delta c} - \frac{d_{T}(\delta + \rho )}{\delta } ) - d_{T} & \rho & - \frac{c(\delta + \rho )}{(1 - \varepsilon )v} \\ \beta ( \frac{s\beta v(1 - \varepsilon )(1 - \eta )}{\delta c} - \frac{d_{T}(\delta + \rho )}{\delta } ) & - (\delta + \rho ) & \frac{c(\delta + \rho )}{(1 - \varepsilon )v} \\ 0 & (1 - \varepsilon )v & - c \end{bmatrix}, \\& J\bigl(P^{*}\bigr) = \begin{bmatrix} \frac{ - \beta d_{T}(\delta + \rho )}{\delta } (R_{0} - 1) & \rho & - \frac{c(\delta + \rho )}{(1 - \varepsilon )v} \\ \frac{\beta d_{T}(\delta + \rho )}{\delta } (R_{0} - 1) & - (\delta + \rho ) & \frac{c(\delta + \rho )}{(1 - \varepsilon )v} \\ 0 & (1 - \varepsilon )v & - c \end{bmatrix}. \end{aligned}$$

*The characteristic equation of equilibrium*$P^{*}$*is*

$$ \lambda ^{3} + \lambda ^{2}P_{1} + \lambda P_{2} + P_{3} = 0, $$

*where*

$$\begin{aligned}& P_{1} = \frac{\beta d_{T} ( \delta + \rho ) ( R_{0} - 1 )}{\delta } + \delta + \rho + c > 0, \\& P_{2} = \beta d_{T} ( \delta + \rho ) ( R_{0} - 1 ) ( c + \delta ) > 0, \\& P_{3} = \beta d_{T}c ( \delta + \rho ) ( R_{0} - 1 ). \end{aligned}$$

*By the Routh–Hurwitz criterion*, *if*

$$ P_{1} > 0,\qquad P_{3} > 0,\quad \textit{and}\quad P_{1}P_{2} - P_{3} > 0. $$

*Finally*, $P^{*}$*is locally asymptotically stable*.

#### Proposition 3.6

*If*$R_{0} > 1$, *then*$P^{0}$*is globally asymptotically stable and unstable otherwise*.

#### Proof

Consider the Lyapunov function

3.2 $$ \begin{aligned}[b] Q\bigl(X^{*},Y^{*},Z^{*} \bigr) &= - \biggl( X^{*}\log \frac{X}{X^{*}} + X^{*} - X \biggr) - \biggl( Y^{*}\log \frac{Y}{Y^{*}} + Y^{*} - Y \biggr) \\ &\quad {}- \biggl( Z^{*}\log \frac{Z}{Z^{*}} + Z^{*} - Z \biggr). \end{aligned} $$

Differentiating (3.2) with respect to “*t*”, we have

3.3 $$\begin{aligned}& \dot{Q} = E\dot{X} + F\dot{Y} + G\dot{Z}, \quad \mbox{where} \\& EX = X^{*} - X, \qquad FY = Y^{*} - Y,\qquad GZ = Z^{*} - Z,\qquad HX = X^{*}, \\& Q = s + E^{2}X(1 - \eta )\beta Z^{*} + F(1 - \eta )\beta XZ + F(1 - \eta )\beta Z^{*}X^{*} \\& \hphantom{Q =}{}+ G \bigl[ (1 - \varepsilon )vY + cZ^{*} \bigr] - sH - E^{2}X(1 - \eta )\beta Z \\& \hphantom{Q =}{}- F^{2}Y(\delta + \rho ) - G(1 - \varepsilon )vY^{*} - cGZ, \quad \mbox{where} \\& Q = A - B, \\& A = s + E^{2}X(1 - \eta )\beta Z^{*} + F(1 - \eta )\beta XZ + F(1 - \eta )\beta Z^{*}X^{*} + G \bigl[ (1 - \varepsilon )vY + cZ^{*} \bigr], \\& B = sH + E^{2}X(1 - \eta )\beta Z + F^{2}Y(\delta + \rho ) + G(1 - \varepsilon )vY^{*} + cGZ. \end{aligned}$$

From (3.3) if $A < B$ then $\dot{Q} \le 0$. Also $\dot{Q} = 0$ iff $X^{*} = X$, $Y^{*} = Y$, $Z^{*} = Z$.

Hence, by [23], $P^{*}$ is globally asymptotically stable when $R_{0} > 1$. □

## Liao’s homotopy analysis method (LHAM)

Let us consider the equation

$$ P\bigl[x(t)\bigr] = 0. $$

We get the following zero-order deformation equation from [22–26]:

4.1 $$ (1 - y)\Im \bigl[\varphi (t;y) - x_{0}(t)\bigr] = yhK(t)P\bigl[\varphi (t;y)\bigr],\quad y \in [0,1],h \ne 0. $$

Here, ℑ is a supplementary linear operator such that $\Im [a_{i}] = 0$ for integral constants $a_{i}$ ($i =1, 2, 3$).

When $y= 0$ and $y = 1$, the zero-order deformation equation can be written as follows:

$$\begin{aligned}& \varphi (t;0) = x_{0}(t), \\& \varphi (t;1) = x(t). \end{aligned}$$

Using the Taylor series expansion of $\varphi (t;y)$ with respect to *y*, we get

4.2 $$ \varphi (t,y) = x_{0}(t) + \sum_{i = 0}^{\infty } x_{i}(t) y^{i}, $$

where

$$x_{i} = \frac{1}{i!}\frac{\partial ^{i}\varphi (t;y)}{\partial y^{i}} \bigg|_{y = 0}. $$

Differentiating the equation with respect to *y* by *i* times, then setting $y = 0$, and lastly dividing them by *i*!, we get the *i*th-order deformation equations:

4.3 $$ \Im \bigl[x_{i}(t) - \psi _{i}x_{i - 1}(t)\bigr] = hK(t)\Re _{i}\bigl[\vec{x}_{{i - 1}}(t)\bigr], $$

where

$$\Re _{i}\bigl[\vec{x}_{{i - 1}}(t)\bigr] = \frac{1}{(i - 1)!}\frac{\partial ^{i - 1}\mathbb{N}[\varphi (t;y)]}{\partial y^{m - 1}} \bigg|_{y = 0} $$

and

$$\psi _{i} = \left \{ \textstyle\begin{array}{c@{\quad}c} 0 & i \le 1 \\ 1 & i > 1 \end{array}\displaystyle \right \} . $$

## Applications

The solution of Eq. (2.1) is defined by using the LHAM method as follows:

5.1 $$\begin{aligned}& \frac{dX}{dt} - s + (1 - \eta )\beta XZ + d_{T}X - \rho Y = 0, \end{aligned}$$

5.2 $$\begin{aligned}& \frac{dY}{dt} - (1 - \eta )\beta XZ + \delta Y + \rho Y = 0, \end{aligned}$$

5.3 $$\begin{aligned}& \frac{dZ}{dt} - (1 - \varepsilon )vY + cZ = 0. \end{aligned}$$

To obtain the analytical solution, the homotopy is

5.4 $$\begin{aligned}& (1 - p) \biggl( \frac{dX}{dt} - s + d_{T}X - \rho Y \biggr) = hp \biggl( \frac{dX}{dt} - s + (1 - \eta )\beta XZ + d_{T}X - \rho Y \biggr), \end{aligned}$$

5.5 $$\begin{aligned}& (1 - p) \biggl( \frac{dY}{dt} + \delta Y + \rho Y \biggr) = hp \biggl( \frac{dY}{dt} - (1 - \eta )\beta XZ + \delta Y + \rho Y \biggr), \end{aligned}$$

5.6 $$\begin{aligned}& (1 - p) \biggl( \frac{dZ}{dt} - (1 - \varepsilon )vY + cZ \biggr) = hp \biggl( \frac{dZ}{dt} - (1 - \varepsilon )vY + cZ \biggr). \end{aligned}$$

Equating $p^{0}$ terms, we get

5.7 $$\begin{aligned}& p^{0}: \biggl( \frac{dX_{0}}{dt} - s + d_{T}X_{0} - \rho Y_{0} \biggr) = 0, \end{aligned}$$

5.8 $$\begin{aligned}& p^{0}: \biggl( \frac{dY_{0}}{dt} + \delta Y_{0} + \rho Y{}_{0} \biggr) = 0, \end{aligned}$$

5.9 $$\begin{aligned}& p^{0}: \biggl( \frac{dZ_{0}}{dt} - (1 - \varepsilon )vY_{0} + cZ_{0} \biggr) = 0. \end{aligned}$$

From Eq. (5.7) ⇒ $X_{0} = \lambda e^{ - d_{T}t} + \lambda _{1}$, where $\lambda = 10^{8} - ( \frac{(s + 10^{ - 2}\rho )}{d_{T}} )$ and $\lambda _{1} = \frac{s + 10^{ - 2}\rho }{d_{T}}$.

From Eq. (5.8) ⇒ $Y_{0} = 10^{ - 2}e^{ - (\delta + \rho )t}$.

From Eq. (5.9) ⇒ $Z_{0} = \lambda _{2}e^{ - ct} + \lambda _{3}$, where $\lambda _{2} = 10 - \frac{10^{ - 2}(1 - \varepsilon )v}{c}$ and $\lambda _{3} = \frac{10^{ - 2}(1 - \varepsilon )v}{c}$.

Again, equating $p^{1}$ terms, we get

5.10 $$\begin{aligned}& \begin{aligned}[b] &p^{1}: \frac{dX_{1}}{dt} + d_{T}X_{1} - \rho Y_{1} - \frac{dX_{0}}{dt} - d_{T}X_{0} + \rho Y_{0} \\ &\hphantom{p^{1}:}\quad = h \biggl( \frac{dX_{0}}{dt} - s + (1 - \eta )\beta X_{0}Z_{0} + d_{T}X_{0} - \rho Y_{0} \biggr), \end{aligned} \end{aligned}$$

5.11 $$\begin{aligned}& \begin{aligned}[b] &p^{1}: \biggl( \frac{dY_{1}}{dt} + \delta Y_{1} + \rho Y{}_{1} \biggr) - \frac{dY_{0}}{dt} - \delta Y_{0} - \rho Y_{0} \\ &\hphantom{p^{1}:}\quad = h \biggl( \frac{dY_{0}}{dt} - (1 - \eta )X_{0}Z_{0} + \delta Y_{0} + \rho Y{}_{0} \biggr), \end{aligned} \end{aligned}$$

5.12 $$\begin{aligned}& \begin{aligned}[b] &p^{1}: \biggl( \frac{dZ_{1}}{dt} - (1 - \varepsilon )vY_{1} + cZ_{1} \biggr) - \frac{dZ_{0}}{dt} - (1 - \varepsilon )vY_{0} + cZ_{0} \\ &\hphantom{p^{1}:}\quad = h \biggl( \frac{dZ_{0}}{dt} - (1 - \varepsilon )vY_{0} + cZ_{0} \biggr). \end{aligned} \end{aligned}$$

From Eq. (5.10) ⇒

$$ \begin{aligned}[b] X_{1} &= \biggl( 10^{8} - \frac{\lambda _{5}}{d_{T} - c} + \frac{\lambda _{6}}{c} - \frac{\lambda _{7}}{d_{T}} + \frac{\rho }{100(d_{T} - \rho - \delta )} \biggr)e^{ - d_{T}t} \\ &\quad {}+ \frac{\lambda _{5}}{d_{T} - c}e^{ - ct} - \frac{\lambda _{6}}{c}e^{ - (c + d_{T})t} + \frac{\lambda _{7}}{d_{T}} - \frac{\rho }{100(d_{T} - \rho - \delta )}, \end{aligned} $$

where $\lambda _{5} = (1 - \eta )\beta \lambda _{1}\lambda _{2}$; $\lambda _{6} = (1 - \eta )\beta \lambda \lambda _{2}$ and $\lambda _{7} = (1 - \eta )\beta \lambda _{1}\lambda _{3} - \lambda _{1}d_{T} - hs$.

From Eq. (5.11) ⇒

$$ Y_{1} = 10^{ - 2} + \lambda _{8}\bigl(1 - e^{ - (d_{T} + c)t}\bigr) + \lambda _{9}\bigl(1 - e^{ - d_{T}t}\bigr) + \lambda _{10}\bigl(1 - e^{ - ct}\bigr), $$

where $\lambda _{8} = \frac{h(1 - \eta )\beta \lambda \lambda _{2}}{\delta + \rho - (d_{T} + c)}$; $\lambda _{9} = \frac{h(1 - \eta )\beta \lambda \lambda _{3}}{\delta + \rho - d_{T}}$ and $\lambda _{10} = \frac{h(1 - \eta )\beta \lambda \lambda _{1}}{\delta + \rho - c}$.

From Eq. (5.12) ⇒

$$ \begin{aligned} Z_{1} &= 10 - \frac{v(1 - \varepsilon )(10^{ - 2} + \lambda _{8} + \lambda _{9} + \lambda _{10})(e^{ - ct} - 1)}{c}\\ &\quad {} - \frac{v(1 - \varepsilon )(e^{ - ct} - e^{ - (d_{T} + c)t})}{d_{T}} + \frac{v(1 - \varepsilon )(e^{ - ct} - e^{ - d_{T}t})}{c - d_{T}} \\ &\quad {}- \frac{10^{ - 2}v(1 - \varepsilon )(1 - h)(e^{ - ct} - e^{ - (\delta + \rho )t})}{c - \delta - \rho } - \bigl( v(1 - \varepsilon ) + c\lambda _{2} \bigr)te^{ - ct}. \end{aligned} $$

The analytical solution of this model using LHAM is

$$ \begin{aligned} X(t) &= \lambda e^{ - d_{T}t} + \lambda _{1} + \biggl( 10^{8} - \frac{\lambda _{5}}{d_{T} - c} + \frac{\lambda _{6}}{c} - \frac{\lambda _{7}}{d_{T}} + \frac{\rho }{100(d_{T} - \rho - \delta )} \biggr)e^{ - d_{T}t} \\ &\quad {}+ \frac{\lambda _{5}}{d_{T} - c}e^{ - ct} - \frac{\lambda _{6}}{c}e^{ - (c + d_{T})t} + \frac{\lambda _{7}}{d_{T}} - \frac{\rho }{100(d_{T} - \rho - \delta )}, \end{aligned} $$

where $\lambda =99\mbox{,}999\mbox{,}900$, $\lambda _{1} = 100$, $\lambda _{5}=4.9995$, $\lambda _{6} = 0.05$, $\lambda _{7} = -1.3$.

5.13 $$\begin{aligned}& \therefore X(t) = 2e^{ - 0.01t} - 0.0746e^{ - 0.67t} - 30, \\& Y(t) = 10^{ - 2}e^{ - (\delta + \rho )t} + 10^{ - 2} + \lambda _{8}\bigl(1 - e^{ - (d_{T} + c)t}\bigr) + \lambda _{9}\bigl(1 - e^{ - d_{T}t}\bigr) + \lambda _{10}\bigl(1 - e^{ - ct}\bigr), \end{aligned}$$

where $\lambda _{8} = -0.0246$, $\lambda _{9} = 2.4627$, $\lambda _{10} = -0.25$.

5.14 $$\begin{aligned}& \therefore Y(t) = 0.01e^{ - 0.07t} + 0.0246e^{ - 0.71t} - 2.4627e^{ - 0.01t} + 0.25e^{ - 0.67t} - 0.2646, \\& Z(t) = \lambda _{2}e^{ - ct} + \lambda _{3} + \biggl( 10 - \frac{(10^{ - 2} + \lambda _{8} + \lambda _{9} + \lambda _{10})(1 - \varepsilon )v}{c} \\& \hphantom{Z(t) =} {}- \frac{(1 - \varepsilon )v}{d_{T}}+ \frac{(1 - \varepsilon )v}{c - d_{T}} - \frac{10^{ - 2}v(1 - \varepsilon )(1 - h)}{c - \delta - \rho } \biggr)e^{ - ct} \\& \hphantom{Z(t) =} {}+\frac{(10^{ - 2}\lambda _{8} + \lambda _{9} + \lambda _{10})v(1 - \varepsilon )}{c} + \frac{(1 - \varepsilon )ve^{ - (d_{T} + c)t}}{d_{T}} \\& \hphantom{Z(t) =} {}- \frac{(1 - \varepsilon )ve^{ - d_{T}t}}{c - d_{T}} + \frac{10^{ - 2}(1 - \varepsilon )v(1 - h)e^{ - (\delta + \rho )t}}{c - \delta - \rho } \\& \hphantom{Z(t) =} {}- \bigl( (1 - \varepsilon )v + (1 + h)c\lambda _{2} \bigr)te^{ - ct}, \end{aligned}$$

where $\lambda _{2}= 9.9990$, $\lambda _{3} = 9.8507$.

5.15 $$ \therefore Z(t) = 0.1e^{ - 0.01t} - 0.0011e^{ - 0.07t} + 16.54e^{ - 0.67t} - 6.6e^{ - 0.68t} + 10.029. $$

## Numerical results

Let us consider the values for numerical results as follows:

$$\begin{aligned}& X_{0}=10^{8},\qquad Y_{0}=10^{-2},\qquad Z_{0}=10, \\& d_{T} = 0.01, \qquad \beta = 1\times 10^{-10},\qquad \delta = 0.07,\qquad v = 100,\\& \eta = 0.5,\qquad \varepsilon = 0.99934,\qquad c = 0.67,\qquad s = 1,\qquad \rho =0. \end{aligned}$$

Let us use Mathematica software to obtain the sixth-order expansions for $X(t)$, $Y(t)$, and $Z(t)$:

6.1 $$\begin{aligned}& \begin{aligned}[b] X(t) &= 100\mbox{,}000\mbox{,}000 + 99\mbox{,}980\mbox{,}234.978ht + 88\mbox{,}734\mbox{,}454.347h^{2}t \\ &\quad {}+ 4\mbox{,}758\mbox{,}843.56h^{3}t + 75\mbox{,}453\mbox{,}444.757h^{4}t + 46\mbox{,}676\mbox{,}678.34h^{5}t \\ &\quad {}+ 92\mbox{,}392.342h^{6}t + 46\mbox{,}347.647h^{2}t^{2} + 637\mbox{,}299.23h^{3}t^{2} + 2326.8293h^{4}t^{2} \\ &\quad {}+ 789.737h^{5}t^{2}+ 6.67889h^{6}t^{2} + 0.63467343h^{3}t^{3} + 2.6646h^{4}t^{3} \\ &\quad {}+ 10.2980376h^{5}t^{3} + 0.9864763638h^{6}t^{3} +\cdots , \end{aligned} \end{aligned}$$

6.2 $$\begin{aligned}& \begin{aligned}[b] Y(t) &= 0.01 + 0.03889431ht + 0.04545347h^{2}t + 0.0845956h^{3}t \\ &\quad {}+0.75743507h^{4}t + 0.043455634h^{5}t + 0.420346045h^{6}t\\ &\quad {} + 0.63242347h^{2}t^{2} + 0.22478453h^{3}t^{2} + 0.18200093h^{4}t^{2}\\ &\quad {}+ 0.700037h^{5}t^{2}+0.6734889h^{6}t^{2}+ 0.00634343h^{3}t^{3}\\ &\quad {} + 0.05464646h^{4}t^{3} + 0.1046667465h^{5}t^{3} + 0.9354378h^{6}t^{3}+ \cdots, \end{aligned} \end{aligned}$$

6.3 $$\begin{aligned}& \begin{aligned}[b] Z(t) &= 10.01 + 620.5464546761ht + 4.4746545h^{2}t + 30.3465436576h^{3}t \\ &\quad {}+2.7346566h^{4}t + 0.05575634h^{5}t + 1.444567045h^{6}t + 5.45656347h^{2}t^{2}\\ &\quad {} + 0.22765803h^{3}t^{2} + 140.74687649h^{4}t^{2} + 45.4455657h^{5}t^{2}\\ &\quad {} +99.645465698h^{6}t^{2} + 80.00698765h^{3}t^{3} + 900.976534646h^{4}t^{3}\\ &\quad {} + 6.196465465h^{5}t^{3} +750.32126378h^{6}t^{3} + \cdots. \end{aligned} \end{aligned}$$

## Error analysis

In this paper, an error analysis has been done to obtain the optimal values of *h*. It is shown in Figs. 8 to 10. The optimum and minimum values of *h* are found from Figs. 11 to 13. For that, we substitute Eqs. (5.1) to (5.3) in (2.1) and get the residual functions, which are shown in what follows. The h value ranges are given in Table 2, and the minimum values are shown in Table 3. Also, the residual errors are calculated in Table 4.

7.1 $$\begin{aligned}& \begin{aligned}[b] ER_{1}(X,Y,Z;h_{1}) &= \frac{d\phi _{X}(t;h_{1})}{dt} - s + (1 - \eta ) \beta _{X}(t;h_{1})_{Z}(t;h_{1})\\ &\quad {} + d_{T X}(t;h_{1}) - \rho _{Y}(t;h_{1}), \end{aligned} \end{aligned}$$

7.2 $$\begin{aligned}& ER_{2}(X,Y,Z;h_{2}) = \frac{d\phi _{Y}(t;h_{2})}{dt} - (1 - \eta ) \beta _{X}(t;h_{2})_{Z}(t;h_{2}) + \delta _{Y}(t;h_{2}) + \rho _{Y}(t;h_{2}), \end{aligned}$$

7.3 $$\begin{aligned}& ER_{3}(X,Y,Z;h_{3}) = \frac{d\phi _{Z}(t;h_{3})}{dt} - (1 - \varepsilon )v_{Y}(t;h_{3}) - c_{Z}(t;h_{3}). \end{aligned}$$

**Table 2:** The *h* value is

*X*(*t*)	−1.3 ≤ *h* ≤ −0.5
*Y*(*t*)	−1.5 ≤ *h* ≤ −0.9
*Z*(*t*)	−1.5 ≤ *h* ≤ −0.9

**Table 3:** The minimum values of $RX(h_{1}^{*})$, $RY(h_{2}^{*})$, $RZ(h_{3}^{*})$

	$h^{*}$	Minimum value
$RX(h_{1})$	−0.877677	3.332434 × 10^−6^
$RY(h_{2})$	−0.649126	2.425675 × 10^−8^
$RZ(h_{3})$	−0.514657	1.57936 × 10^−12^

**Table 4:** The residual errors for $ER_{1}$, $ER_{2}$, and $ER_{3}$ for $t \in (0,1)$

*t*	${ER} _{1}(X,Y,Z;h_{1}^{*})$	${ER} _{2}(X,Y,Z;h_{2}^{*})$	${ER} _{3}(X,Y,Z;h_{3}^{*})$
0.0	7.677888 × 10^−6^	5.536737 × 10^−5^	3.646747 × 10^−8^
0.1	3.647548 × 10^−3^	9.745774 × 10^−6^	8.747839 × 10^−9^
0.2	5.566774 × 10^−2^	2.747899 × 10^−4^	4.889889 × 10^−6^
0.3	1.647689 × 10^−5^	6.738893 × 10^−3^	9.374839 × 10^−7^
0.4	9.673487 × 10^−9^	1.988787 × 10^−4^	7.838439 × 10^−6^
0.5	6.674639 × 10^−8^	7.889987 × 10^−6^	2.473748 × 10^−5^
0.6	9.782357 × 10^−7^	3.774898 × 10^−5^	1.838493 × 10^−7^
0.7	6.646388 × 10^−1^	8.738783 × 10^−4^	5.737888 × 10^−6^
0.8	2.663879 × 10^−6^	1.789888 × 10^−6^	6.737882 × 10^−4^
0.9	8.653467 × 10^−4^	7.893289 × 10^−7^	7.374378 × 10^−9^
1	4.778898 × 10^−5^	4.789897 × 10^−6^	3.747889 × 10^−5^

Let us consider the square residual error for sixth-order approximation [27, 28]:

7.4 $$\begin{aligned}& RX(h_{1}) = \int _{0}^{1} \bigl(ER_{1}(X,Y,Z;h_{1}) \bigr)^{2}dt, \end{aligned}$$

7.5 $$\begin{aligned}& RY(h_{2}) = \int _{0}^{1} \bigl(ER_{2}(X,Y,Z;h_{2}) \bigr)^{2}dt, \end{aligned}$$

7.6 $$\begin{aligned}& RZ(h_{3}) = \int _{0}^{1} \bigl(ER_{3}(X,Y,Z;h_{3}) \bigr)^{2}dt. \end{aligned}$$

The minimal values of $RX(h_{1})$, $RY(h_{2})$, $RZ(h_{3})$ are as follows:

$$ \frac{dRX(h_{1}^{*})}{dh_{1}} = 0,\qquad \frac{dRY(h_{2}^{*})}{dh_{2}} = 0,\qquad \frac{dRZ(h_{3}^{*})}{dh_{3}} = 0. $$

We consider the optimal values of $h_{1}^{*}$, $h_{2}^{*}$, and $h_{3}^{*}$ for all of the cases:

$$ h_{1}^{*} = -0.877677,\qquad h_{2}^{*} = -0.649126,\qquad h_{3}^{*} = -0.514657. $$

## Discussion

**Figure 2:**
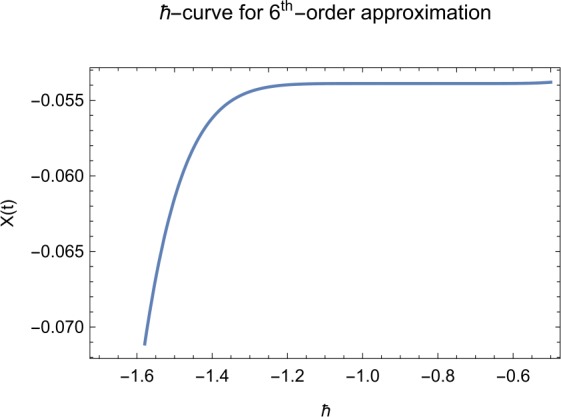
The *h*-curves of sixth-order approximations for $X(t)$

**Figure 3:**
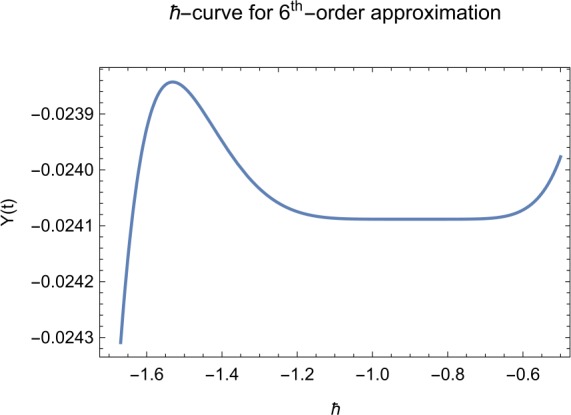
The *h*-curves of sixth-order approximations for $Y(t)$

**Figure 4:**
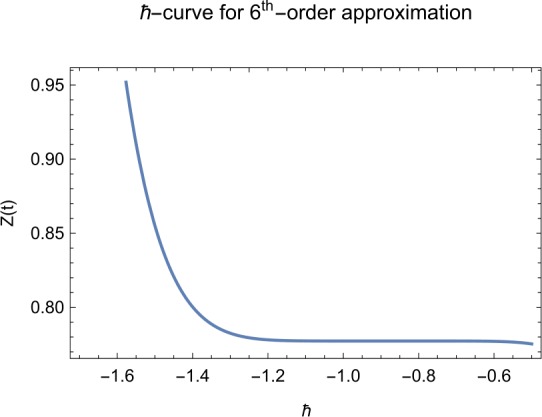
The *h*-curves of sixth-order approximations for $Z(t)$

**Figure 5:**
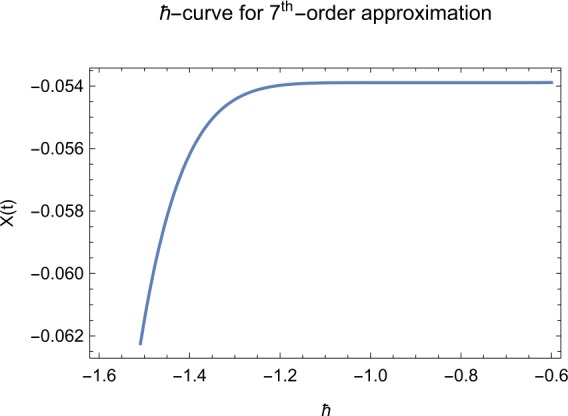
The *h*-curves of seventh-order approximations for $X(t)$

**Figure 6:**
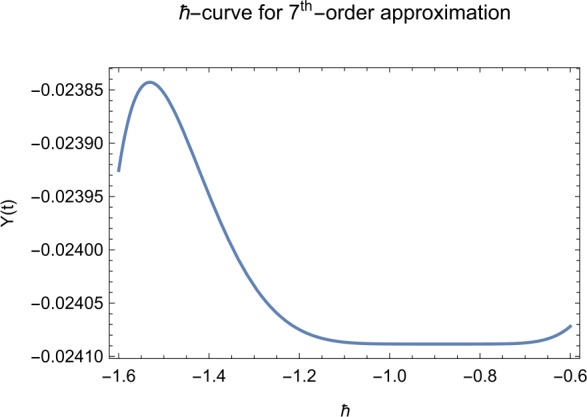
The *h*-curves of seventh-order approximations for $Y(t)$

**Figure 7:**
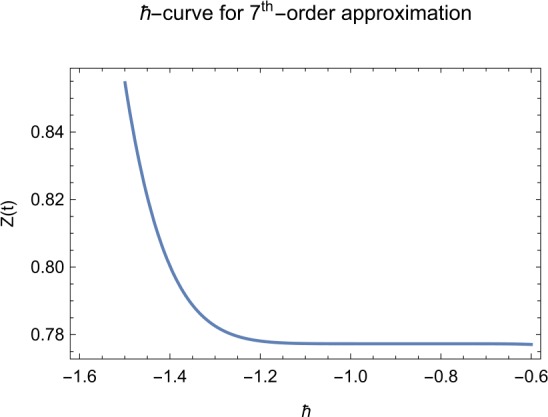
The *h*-curves of seventh-order approximations for $Z(t)$

**Figure 8:**
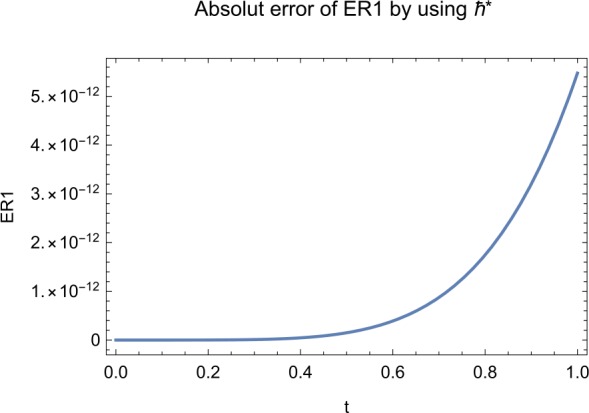
The residual error function of Eq. (7.1)

**Figure 9:**
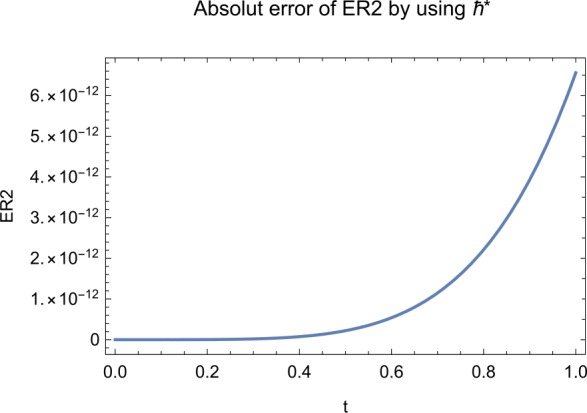
The residual error function of Eq. (7.2)

**Figure 10:**
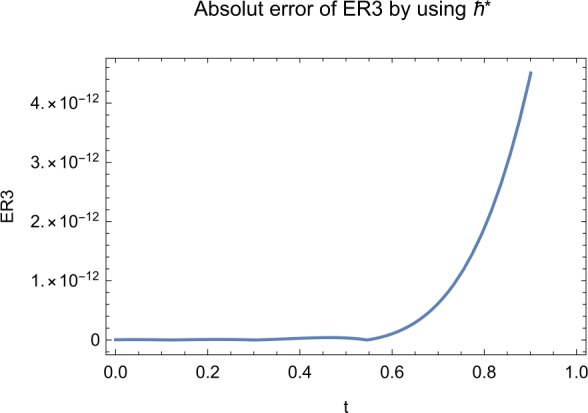
The residual error function of Eq. (7.3)

**Figure 11:**
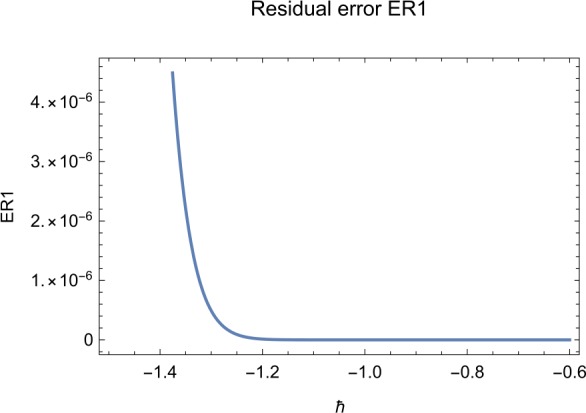
The optimum and minimum values of $X(t)$

**Figure 12:**
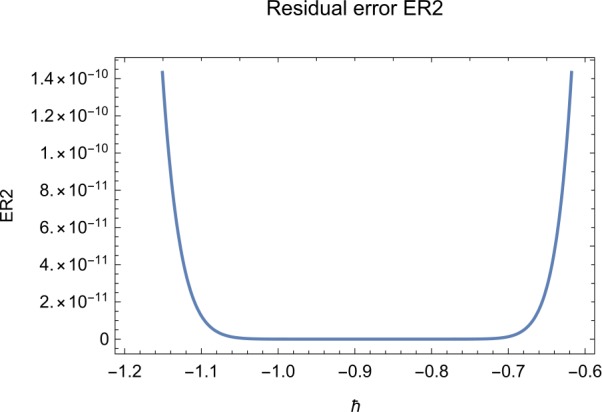
The optimum and minimum values of $Y(t)$

**Figure 13:**
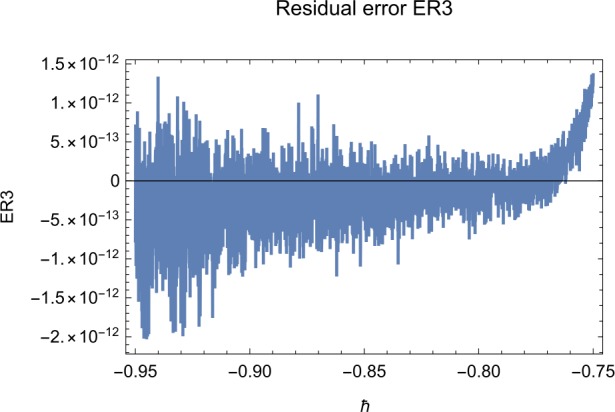
The optimum and minimum values of $Z(t)$

We found the solution from (5.13)–(5.15), which contain ‘*h*’ that shows an easy technique to control and adjust curves to confirm series solution to converge, which is recommended by Liao [25, 26]. Figures 2–7 show the plots of sixth and seventh term approximation of $X(t)$, $Y(t)$, and $Z(t)$. Through these curves, it is clear that the valid region of ‘*h*’ is parallel to the horizontal axis. The valid region is listed in Table 2. Figures 8–13 show the residual error function of Eqs. (7.1), (7.2), and (7.3) using the sixth-order approximate solution for the different values of $h = -1.3$, $h = -1.5$, and $h = -0.9$.

## Conclusion

Hepatitis B virus is proved to be a dangerous disease affecting people enormously. Though there has been vaccine to cure this disease, the antiviral therapy is recognized as the best method to eradicate this disease utmost at the root level. In this paper, the reproduction number $R_{0}$ is used to find the global dynamics. If $R_{0} \le 1$, the disease-free equilibrium is globally asymptotically stable. Furthermore, if $R_{0} > 1$, the endemic equilibrium is globally asymptotically stable. The potentiality of HAM depicts the convergence of sequence solution for nonlinear differential equations, which we proved in this paper confirming that HAM is a very effective and powerful technique to find the approximate semi-analytical solutions. The numerical simulations have been obtained up to sixth-order approximations, and error analysis has been done with the help of Mathematica software. The study of mathematical models of disease development will allow better knowledge of disease evolution to reduce the incidence of accidental infections among healthcare professionals and to improve the quality of life of patients who may be given therapies already experienced in other hepatitis [29]. This research paper can be a framework for the young researchers to do a further research and design an effective antiviral therapy and drug design.
